# Optimizing Athletic Performance: A Systems Framework for Adaptive Training, Load Management, and Decision-Making

**DOI:** 10.3390/jfmk11030245

**Published:** 2026-06-23

**Authors:** Dan Cristian Mănescu, Cristina Filip, Cristina Ionela Nae, Rela Valentina Ciomag

**Affiliations:** Department of Sport and Physical Education, Bucharest University of Economic Studies, 010374 Bucharest, Romania; dan.manescu@defs.ase.ro (D.C.M.); rela.ciomag@defs.ase.ro (R.V.C.)

**Keywords:** athlete monitoring, training load, load management, readiness, recovery, neuromuscular fatigue, adaptive training, performance optimization, decision-making, strength and conditioning

## Abstract

Although athlete monitoring can quantify training exposure and athlete status with increasing detail, conversion into daily training decisions remains inconsistent. This structured narrative review synthesizes evidence on training load, neuromuscular readiness, recovery, fatigue interpretation, measurement reliability, applied decision-making, and proposes the LOAD-R framework: a systems model linking Load, Organism response, Adaptive state, Decision, and Re-evaluation. A transparent non-PRISMA strategy was used because the aim was conceptual integration and framework development rather than effect-size pooling. Evidence was organized around field-applicable monitoring domains and their decision value. LOAD-R builds on existing monitoring approaches by organizing single indicators, fixed thresholds, and dashboard alerts into an explicit interpretation-to-action sequence. It classifies athlete state into adaptive, functional-overload, underloaded, uncertain, or maladaptive zones, each linked to progress, maintain, modify, deload, or recover decisions. The framework also provides implementation levels and testable predictions. By framing monitoring as adaptive decision support rather than passive data collection, LOAD-R may improve decision consistency, reduce maladaptive training responses, and enhance the practical value of athlete monitoring in applied sport settings.

## 1. Introduction

Contemporary athlete monitoring has moved from specialist laboratories into routine coaching practice. External-load systems, session rating of perceived exertion (sRPE), wellness questionnaires, heart-rate-derived markers, countermovement-jump (CMJ) testing, and wearable technologies can now describe both training exposure and athlete response with high temporal density. Yet measurement capacity is not the same as decision quality: monitoring improves practice only when it changes what coaches do, when they do it, and how strongly they adjust training [1,2,3,4,5,6,7,8,9,10,11,12,13,14].

This distinction is central because adaptation depends on the interaction between the imposed stimulus and the athlete’s current response capacity. The same workload may be productive, insufficient, or excessive according to sleep, soreness, autonomic status, recent load trajectory, injury history, competitive phase, and psychological or environmental stress. The same readiness score may also imply different actions in maximal-strength, sprint-power, endurance, or congested team-sport contexts [15,16,17,18,19,20,21,22].

In applied settings, monitoring data are usually managed through a recurring workflow. External-load information is captured during or immediately after training; internal-load and session-difficulty information are commonly collected shortly after the session; readiness, wellness, sleep, soreness, and autonomic markers are often reviewed before the next training exposure or on the following morning; and performance quality is checked during key sessions or across the microcycle. These signals are then interpreted against individual baselines, measurement reliability, recent load trajectory, injury or illness status, competition timing, and coach observations before being translated into training modification. The weak point is often not data collection itself, but the lack of an explicit rule for combining these layers, selecting a proportional action, and re-evaluating whether that action improved readiness, performance, or training tolerance.

A further limitation is interpretative fragmentation. Training load, fatigue, readiness, and injury risk are often interpreted through isolated indicators, such as week-to-week load changes or acute-to-chronic workload ratios. Such tools can summarize exposure, but they become weaker when used as universal thresholds, detached from reliability, measurement error, context, and individual response. Dashboard alerts can also multiply signals without establishing an action hierarchy. The field may therefore benefit from a systems architecture that supports decisions without creating false precision [23,24,25,26,27,28,29,30,31,32,33,34,35].

### Research Questions and Aims

To address these gaps, this structured narrative review is guided by six interrelated questions:

**Q1.**  Which external and internal load variables are most useful for describing the training stimulus in applied sport settings?
**Q2.**  Which readiness, fatigue, recovery, and performance markers are most applicable for field-based decision-making?
**Q3.**  Why does athlete monitoring frequently fail to translate into explicit training actions?
**Q4.**  How can load, organism response, adaptive state, decision-making, and re-evaluation be integrated into a coherent systems model?
**Q5.**  Which practical decision rules can guide training progression, maintenance, modification, deloading, or recovery emphasis?
**Q6.**  Which testable predictions emerge from a systems-based model of adaptive training and load management?

The aim of this review is therefore not to produce another catalog of monitoring tools. Its purpose is to synthesize the applied sport-science literature into a practical framework—the LOAD-R model—that connects training load, athlete response, adaptive-state classification, training decisions, and repeated re-evaluation. The specific contribution is to position athlete monitoring as a decision-support process rather than as a data-collection process.

Accordingly, the novelty claimed here is organizational and translational rather than physiological: LOAD-R integrates established monitoring principles into a coach-facing sequence that makes the transition from data interpretation to training action explicit, auditable, and revisable.

## 2. Methodological and Conceptual Framework

### 2.1. Methodological Approach

This article was designed as a structured narrative review with a conceptual-framework component. This format was selected because the question spans heterogeneous evidence domains and the intended output is an integrative decision model, not a pooled effect estimate. The review followed established narrative-review quality principles: clear aim, transparent search logic, relevance-based selection, critical synthesis, and explicit conceptual contribution [15].

Literature was searched iteratively in PubMed/MEDLINE, Scopus, Web of Science, SPORTDiscus, and Google Scholar. Searches prioritized work published from January 2010 to April 2026; however, foundational pre-2010 sources were retained when they were necessary for historical or conceptual positioning. Terms were combined across six blocks: training load, athlete monitoring, internal response, neuromuscular readiness, recovery, and decision-making. Key reference lists were also screened.

The decision to prioritize 2010–2026 evidence was intended to capture contemporary athlete-monitoring practice; pre-2010 sources were included where they established key concepts such as fitness-fatigue modeling, perceived exertion, and session-RPE monitoring. Recent 2025–2026 sources were retained only when directly relevant to the manuscript’s conceptual domains and were not used as substitutes for foundational literature.

Evidence was charted against predefined translational themes: external and internal load; athlete-reported status; neuromuscular readiness; autonomic and sleep-related recovery; workload-injury and workload-performance interpretation; reliability and individual response; and field implementation. For each domain, attention was given to study type, athlete population, variables, outcomes, measurement constraints, and actionability.

Inclusion criteria were: (1) relevance to athletes, trained individuals, team sports, individual sports, or structured performance settings; (2) discussion or reporting of load, readiness, recovery, fatigue, performance, injury risk, or adaptation; (3) implications for monitoring interpretation or training prescription; and (4) sufficient detail to inform a decision framework.

Exclusion criteria were clinical rehabilitation without athletic-performance context, physical-activity studies without training relevance, technology-validation papers disconnected from action, animal or molecular-only studies without sport-relevant mechanisms, and papers describing tools without interpretation or decision value.

Study selection proceeded through title/abstract screening, full-text conceptual screening, and backward/forward citation tracking. Because this was a narrative review, the purpose was not exhaustive retrieval, PRISMA flow reporting, formal risk-of-bias scoring, or meta-analysis. The non-PRISMA approach was selected to support conceptual integration across heterogeneous evidence. This approach is appropriate for conceptual synthesis in applied sport science, where evidence is distributed across heterogeneous study designs, monitoring technologies, athlete populations, and outcome measures. Rather than estimating a pooled effect, the objective was to identify convergent principles that could support field-based interpretation and decision-making. Accordingly, studies were not treated as interchangeable units of evidence, but were weighted according to conceptual relevance, ecological validity, methodological quality, and practical actionability.

The iterative search and screening process identified approximately 280–320 potentially relevant records across databases and citation tracking sources. After title/abstract and full-text conceptual screening, approximately 110–140 articles were retained for synthesis based on relevance and decision-making applicability.

Evidence weighting was descriptive and decision-oriented. Consensus statements, systematic reviews, longitudinal monitoring studies, intervention studies, and reliability papers were given greater weight when they directly informed decision logic; cross-sectional, technology-validation, and expert-practice papers were used mainly to clarify measurement feasibility, implementation constraints, or conceptual positioning. No study was treated as providing universal thresholds across all sports.

A representative search strategy included combinations such as: (“training load” OR “athlete monitoring”) AND (“fatigue” OR “readiness” OR “recovery”) AND (“decision-making” OR “performance”).

To improve reproducibility, database-specific search templates were added as follows: PubMed/MEDLINE: (“training load” OR “athlete monitoring” OR “session RPE”) AND (fatigue OR readiness OR recovery OR performance) AND (athlete* OR sport*); Scopus: TITLE-ABS-KEY((“training load” OR “athlete monitoring” OR “internal load” OR “external load”) AND (fatigue OR readiness OR recovery OR “decision-making”) AND (athlete OR sport OR “team sport”)); Web of Science: TS = ((“training load” OR “athlete monitoring” OR “readiness monitoring”) AND (fatigue OR recovery OR performance) AND (sport OR athlete)); SPORTDiscus: (“training load” OR “session RPE” OR “athlete monitoring”) AND (readiness OR fatigue OR recovery OR “load management”); Google Scholar: “athlete monitoring” “training load” readiness recovery “decision-making”; the first 100 results were screened because relevance declined substantially beyond that point.

The descriptive retrieval log was: PubMed/MEDLINE, *n* = 64; Scopus, *n* = 86; Web of Science, *n* = 72; SPORTDiscus, *n* = 43; Google Scholar, *n* = 100 screened records; and backward/forward citation tracking, *n* = 31. After duplicate removal, approximately 282 unique records were screened at the title/abstract level; 164 sources were assessed in full-text or full conceptual screening; and 139 sources were retained in the final reference list after the reviewer-requested additions. These numbers are reported to improve transparency, but they should not be interpreted as a PRISMA flow diagram or as evidence of exhaustive retrieval.

While the review was not designed as a systematic or exhaustive retrieval, emphasis was placed on relevance to decision-making, ecological validity, and translational value.

These choices mean that the synthesis is reproducible at the level of search logic, selection rationale, and conceptual weighting, but not in the same way as a systematic review with registered protocol, duplicate screening, and formal risk-of-bias scoring.

### 2.2. Conceptual Contribution and Positioning

The conceptual contribution is deliberately framed as translational rather than entirely de novo: LOAD-R reorganizes established athlete-monitoring concepts into an explicit decision architecture. LOAD-R is not proposed as a new physiological law, universal threshold system, or replacement for coaching expertise. It links five elements often treated separately: imposed load, organism-level response, inferred adaptive state, training decision, and subsequent re-evaluation.

Relative to existing approaches, LOAD-R adds four decision-facing elements. Compared with fitness-fatigue models, it emphasizes daily field interpretation rather than retrospective performance modeling. Compared with acute-to-chronic workload approaches, it avoids stand-alone load-ratio thresholds and interprets load through response capacity, trajectory, and measurement error. Compared with dashboards, it converts convergent signals into action categories. Compared with generic readiness monitoring, it requires each decision to be checked against subsequent outcomes and used to update the athlete's baseline [16,17,18,19,20].

This distinction is important because prior work already supports individualized monitoring, contextual interpretation, and decision-support logic. LOAD-R is intended to make its sequence operational by requiring each monitoring signal to pass through reliability appraisal, individual baseline comparison, contextual interpretation, action selection, and outcome re-checking before it influences training prescription.

Consequently, the manuscript does not claim that LOAD-R is empirically superior to earlier frameworks at this stage. Its claim is limited to synthesis, operational sequencing, and decision transparency: the model specifies how existing monitoring concepts can be linked to auditable training actions and then tested empirically.

More specifically, existing monitoring cycles emphasize collecting, interpreting, and applying data, but they often leave the action boundary implicit. Fitness-fatigue and performance-modeling approaches describe the relationship between training input and performance output, but they are frequently retrospective or parameter-estimation oriented. Acute-to-chronic workload approaches summarize recent exposure, but they do not by themselves explain whether a given athlete should progress, maintain, modify, deload, or recover on a specific day. Readiness dashboards increase visibility, but may present multiple signals without a hierarchy for reliability, redundancy, context, and outcome checking. LOAD-R therefore advances current practice primarily by converting these established ideas into a state-action-re-evaluation sequence rather than by proposing a new biomarker, ratio, or physiological mechanism.

Its practical advantages are explicitness and auditability: each decision can be traced back to the stimulus, athlete response, adaptive-state interpretation, selected action, and subsequent outcome. Its limitations are equally important: the framework requires local calibration, reliable measurements, sport-specific variable selection, practitioner expertise, and empirical validation before it can be treated as superior to alternative monitoring workflows.

**Figure 1:**
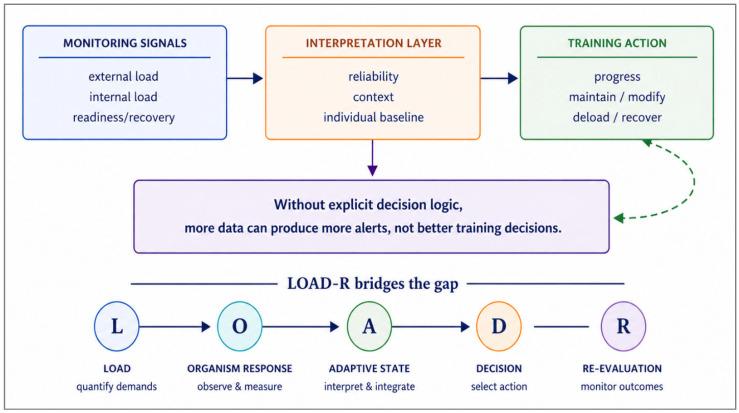
The monitoring–decision gap in athletic performance. Modern monitoring expands measurement capacity, but without an explicit interpretation and decision layer, information density can increase uncertainty rather than improve training action [1,2,3,4,5,6,7,8,9,10,11,12,13,14,15].

Despite the rapid expansion of athlete-monitoring technologies, the translation of data into consistent and actionable training decisions remains limited. This disconnect between measurement and decision-making represents a central challenge in applied sport science. Figure 1 illustrates this monitoring–decision gap.

The LOAD-R framework was developed to address this gap. Its intended contribution lies not in introducing new monitoring variables, but in structuring a decision-oriented architecture that explicitly connects measurement, interpretation, and action.

Unlike existing approaches that emphasize quantification (e.g., load metrics) or description (e.g., readiness status), LOAD-R formalizes one possible transition from data to decision by integrating adaptive-state classification and feedback-driven re-evaluation into a single operational loop.

In this sense, the framework aims to reposition athlete monitoring as a decision-support process rather than a data-collection process, with explicit links between observed signals and training actions.

To operationalize the LOAD-R framework in applied settings, Table 1 organizes the major evidence domains according to their specific role in the decision-making process.

## 3. Closing the Monitoring–Decision Gap: Interpreting Data for Action

Monitoring often fails to improve decisions because metrics describe different layers of the training process. External-load variables quantify mechanical or task exposure but not how costly the stimulus was for a given athlete. Internal-load variables describe perceived or physiological strain, but can also reflect sleep, stress, nutrition, illness, emotional load, or environmental conditions. Either domain can mislead when interpreted alone [36,37,38,39,40].

This limitation is magnified in team sports and mixed-method training environments. The same drill can impose different sprint, acceleration, contact, and cognitive demands across players, while similar sRPE values may follow very different mechanical exposures. The relevant question is therefore not whether external or internal load is superior, but whether their convergence, mismatch, and trajectory justify a specific training action [41,42,43,44,45,46].

Dashboard inflation can further widen the monitoring–decision gap. When many variables are displayed as traffic-light alerts, practitioners may receive signals without hierarchy. A suppressed marker may reflect true fatigue, normal biological variability, poor testing conditions, or measurement noise. LOAD-R therefore prioritizes signal relevance, trajectory, measurement reliability, and actionability over data abundance; convergence is useful only when markers are reliable, sufficiently non-redundant, and aligned with the sport-specific question.

### The Practical Meaning of Internal-External Load Mismatch

Internal-external mismatch is a high-value decision signal when interpreted cautiously. Normal external load with elevated internal strain may indicate hidden fatigue, stress, sleep debt, early illness, poor fueling, or environmental intolerance. High external load with stable internal strain and preserved output may indicate improved tolerance. High load plus high internal strain, suppressed readiness, and declining output suggest the need for protection and recovery [46,47,48,49,50,51,52,53,54,55,56,57,58].

This logic does not remove coaching judgment; it structures it. Practitioners still interpret signals in relation to the training goal, competitive phase, athlete history, and session priority, but the path from observation to action becomes explicit and auditable.

**Figure 2:**
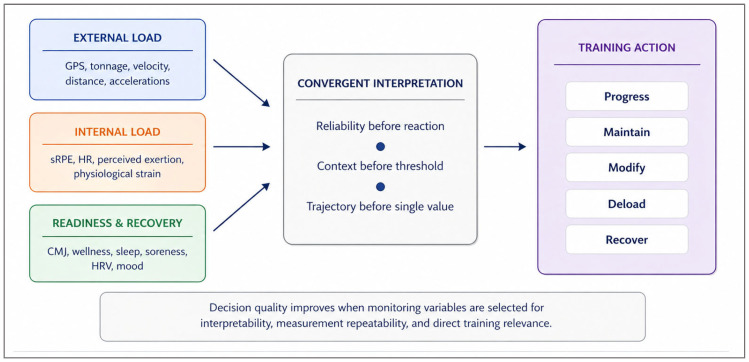
Evidence-to-decision architecture. External load, internal load, and readiness markers should be interpreted through convergent logic before they are converted into training actions. The figure emphasizes reliability, noise, and multi-marker agreement as intermediate steps [36,37,38,39,40,41,42,43,44,45,46,47,48,49,50,51,52,53,54,55,56,57,58].

Because external exposure, internal strain, and readiness markers each capture only part of the training process, Figure 2 shows how evidence is filtered through reliability, convergence, and context before becoming a decision.

Following the evidence-to-decision pathway in Figure 2, Table 2 translates monitoring domains into field-applicable variables, interpretations, and minimum implementation requirements.

Terminology in Table 2 distinguishes raw perceived exertion from derived session-load indices. RPE or perceived exertion refers to the athlete’s rating of effort; session RPE describes the post-session RPE score; sRPE-load refers to session RPE multiplied by session duration; and session difficulty can capture a broader qualitative appraisal of how hard or complex the session felt.

This section establishes the interpretative layer required before any LOAD-R decision is made.

## 4. Monitoring Domains for Adaptive Training

External load: stimulus. External load represents the work performed by the athlete, including distance, high-speed running, accelerations, decelerations, sprint exposure, collisions, resistance-training tonnage, velocity loss, session duration, and training density. It is essential because adaptation requires exposure, but it should be interpreted as stimulus information rather than direct evidence of adaptation [36,37,38,39,40,41,42,43,44,45,59,60,61,62,63,64].

Internal load: the cost of the stimulus. Internal load is the athlete’s physiological and perceptual response to training. RPE-based monitoring remains valuable because it is inexpensive, scalable, and field-compatible, but RPE, session RPE, and sRPE-load should not be used interchangeably. RPE denotes perceived effort, session RPE is usually collected after the session, and sRPE-load is the product of session RPE and session duration. Heart-rate-derived methods add physiological resolution, especially in endurance and conditioning contexts, but may miss high-intensity neuromuscular or contact demands. Subjective measures should therefore be treated as athlete-response data rather than as weak substitutes for technology [46,47,48,49,50,51,52,53,54,65,66,67,68,69,70,71,72,73,74,75,76,77].

In non-professional or low-resource settings, subjective scales require athlete familiarization, clear anchoring, repeated instructions, and periodic checks against observable outputs. When athletes are inexperienced with RPE, the variable should be down-weighted and interpreted with coach observations, session duration, and simple performance markers rather than used as a standalone decision trigger.

Readiness and neuromuscular status. Neuromuscular readiness is especially relevant for speed, power, strength, and collision sports. CMJ-derived variables can detect fatigue or rebound when protocols are standardized and interpreted against individual baselines. In LOAD-R, convergence should be assessed through predefined within-athlete rules rather than impression alone: for example, two or more reliable markers moving beyond expected day-to-day variation in the same direction, supported by the session objective and context. Readiness should therefore not be reduced to a single jump score or to a simple majority vote; interpretation is strongest when complementary neuromuscular, perceptual, contextual, and performance indicators point toward the same practical action [78,79,80,81,82,83,84,85,86,87,88,89,90,91,92,93,94,95,96,97,98].

Recovery, sleep, and contextual load. Recovery determines whether a subsequent load is likely to be decoded as adaptation or overload. Sleep duration and quality, soreness, mood, appetite, psychological stress, travel, academic or occupational demands, and illness symptoms can all modify training tolerance. In dual-career athletes and university sport, these contextual loads may be decisive because training is only one component of total stress [55,56,57,58,59,60,61,62,63,64,65,66,67,68,69,70,71,72].

## 5. The LOAD-R Framework

LOAD-R is organized around five linked components: Load, Organism response, Adaptive state, Decision, and Re-evaluation. The model begins by defining the intended and actual training stimulus, interprets athlete response across multiple channels, classifies the temporary adaptive state, selects a proportional training action, and checks the outcome against subsequent readiness and performance [16,17,18,19,20,21,22,73,74,75,76,77,78,79,80,81,82,83,84,85,86,87,88].

**Figure 3:**
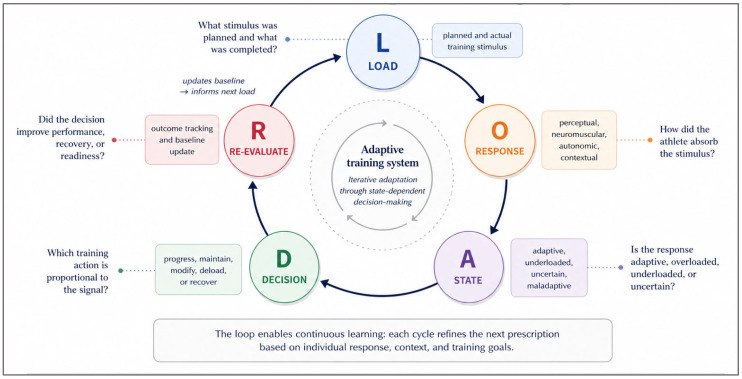
The LOAD-R adaptive training framework. Athletic performance optimization is conceptualized as a repeated loop in which training load is interpreted through athlete response, converted into an adaptive-state classification, translated into action, and re-evaluated over time [1,2,3,4,5,6,7,8,9,10,11,12,13,14,15,16,17,18,19,20,21,22,73,74,75,76,77,78,79,80,81,82,83,84,85,86,87,88]. The figure extends the evidence-to-decision pathway by embedding it within a repeated adaptive loop. The arrows indicate the sequential progression through the LOAD-R cycle, whereas the dotted central circle represents the continuous adaptive feedback process linking all framework components.

Building on load-response, periodization, readiness, and decision-support evidence, Figure 3 presents LOAD-R as a repeated adaptive loop rather than a static monitoring dashboard.

### 5.1. Load

The first step is to define what the athlete was exposed to: the intended session objective, the actual completed load, and the difference between planned and realized work. This prevents readiness from being interpreted without reference to the stimulus that preceded it [1,2,3,4,5,6,7,8,9,10,11,12,13,14,36,37,38,39,40,41,42,43,44,45].

### 5.2. Organism Response

The second step is to interpret how the athlete absorbed the stimulus. The term “organism response” is used because training tolerance is perceptual, autonomic, neuromuscular, cognitive, psychological, and contextual, not only muscular or cardiovascular [46,47,48,49,50,51,52,53,54,55,56,57,65,66,67,68,69,70,71,72,73,74,75,76,77].

### 5.3. Adaptive State

The third step is classification. The athlete should not be classified in a binary ready/not-ready manner. More useful temporary categories include adaptive tolerance, functional overload, maladaptive stress, underload, and uncertainty, interpreted relative to individual baselines, measurement error, and training objective [89,90,91,92,93,94,95,96,97,98].

### 5.4. Decision

The fourth step is the training action. LOAD-R uses five default decisions: progress, maintain, modify, deload, or recover. The decision should be proportional to the strength, reliability, independence, and practical relevance of the signal, rather than triggered automatically by a single marker or by a redundant set of markers [11,23,24,25,26,27,28,29,30,31,32,33,34,35,89,90,91,92,93,94,95,96,97,98].

### 5.5. Re-Evaluation

The final step is re-evaluation. If performance rebounds after a deload, accumulated fatigue was likely present. If suppression persists, practitioners should reassess illness, injury, sleep, nutrition, psychological load, or program design. Re-evaluation also updates the rolling baseline [94,95,96,97,98].

### 5.6. Handling Measurement Error in Daily Decisions

Because daily monitoring values contain noise, LOAD-R decisions should begin with measurement-error appraisal rather than raw change detection. A practical sequence is: (1) establish an individual baseline under stable testing conditions; (2) quantify typical error, coefficient of variation, SEM, or ICC for the selected marker; (3) define a minimum decision threshold; (4) grade the signal according to whether it exceeds expected error; and (5) re-check the outcome before updating the athlete baseline [91,92].

For low-resource field use, a simple minimum-detectable-change rule is preferable to reacting to every fluctuation: changes within typical error or SEM support maintain/retest decisions; one reliable marker exceeding error supports a cautious modify/check-context decision; repeated changes or two complementary markers exceeding error in the same practical direction support stronger progress, deload, or recover decisions. Where sufficient longitudinal data exist, Bayesian or probabilistic updating can refine these rules, but the conservative default is that no high-cost decision should be triggered by a single value that remains inside measurement error.

When SEM is available, the minimum detectable change can be expressed as MDC95 = 1.96 × sqrt(2) × SEM. In LOAD-R, values below this boundary are treated as uncertain unless supported by context, repeated testing, or a relevant performance outcome; values above this boundary are interpreted in relation to session goal, athlete history, and marker specificity rather than as automatic stop/go rules.

The operational questions, primary data sources, and default decision relevance for each LOAD-R component are summarized in Table 3.

## 6. Decision Zones and Training Actions

Decision-zone logic is the practical bridge between monitoring and action. Decisions should be based on the interaction between recent load, readiness, output, and context rather than on isolated variables. A high load with stable readiness and improving output differs from a high load with suppressed readiness and declining output; high readiness after low-load days may signal under-stimulation [23,24,25,26,27,28,29,30,31,32,33,34,35,89,90,91,92,93,94,95,96,97,98].

**Figure 4:**
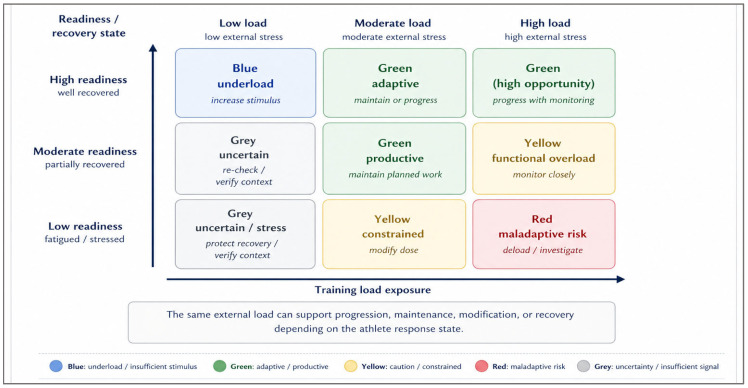
Decision-zone matrix for adaptive training. The same load can imply progression, maintenance, protection, recovery, or contextual reassessment depending on the athlete-readiness state and recent trajectory. Color coding reflects decision zones ranging from underload (blue) to maladaptive risk (red), with gray indicating uncertainty requiring reassessment [23,24,25,26,27,28,29,30,31,32,33,34,35,89,90,91,92,93,94,95,96,97,98].

Drawing on workload-injury debates and decision-support principles, Figure 4 presents decision zones as a practical matrix rather than fixed universal thresholds.

These zones are heuristic labels for communication and audit, not universal diagnostic categories. Their thresholds, time windows, and marker combinations must be defined locally according to sport demands, measurement reliability, competitive calendar, and athlete history.

Because Figure 4 is intended to guide action rather than classification alone, Table 4 defines each zone, its typical interpretation, default action, and risk if misread.

To convert the same zone logic into coach-facing rules, Table 5 presents conditional if-then adjustments that preserve context and individual judgment.

## 7. Athlete Response Archetypes and Individualization

Individualization is essential because athletes do not respond identically to comparable training. LOAD-R therefore interprets monitoring data through temporary response archetypes. These are not diagnostic labels or fixed traits; they are practical patterns that help staff identify whether an athlete is adapting, accumulating fatigue, receiving insufficient stimulus, or carrying contextual stress.

**Figure 5:**
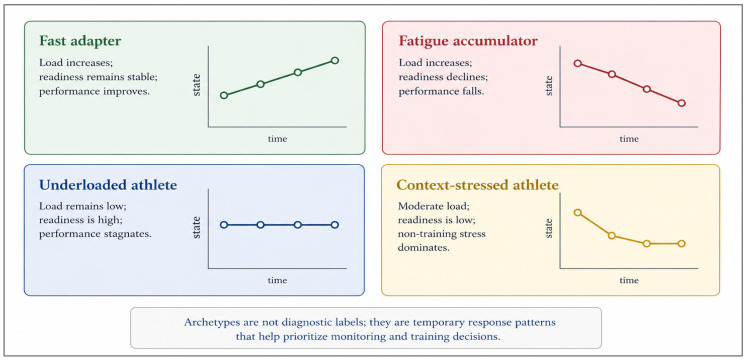
Athlete response archetypes under comparable training load. Similar external stimuli can produce different response trajectories depending on recovery capacity, contextual stress, neuromuscular state, and recent adaptation history [99,100,101,102,103,104,105,106,107,108,109,110,111,112,113,114,115,116,117,118,119,120,121,122].

Given evidence that athletes respond heterogeneously to comparable loads, Figure 5 illustrates response archetypes that may occur under the same external stimulus.

Table 6 extends the archetype logic into monitoring priorities and coaching responses so that individualization remains observable and actionable.

## 8. Field Implementation Across Resource Levels

Implementation should scale to available resources. A university or amateur team can apply LOAD-R with a training diary, RPE, or sRPE-load, a short wellness questionnaire, and simple jump or sprint testing. A professional team can add GPS, force-plate CMJ, HRV, sleep monitoring, and integrated dashboards. Decision quality depends less on technology volume than on reliable measurement, consistent interpretation, and predefined actions.

Because implementation quality should not depend on expensive technology alone, Table 7 outlines scalable LOAD-R configurations across resource levels.

At low-cost and non-professional levels, implementation should begin with education and calibration of subjective scales. Staff should explain anchors, collect repeated values in stable conditions, and compare athlete reports with observable training quality before attaching high decision weight to self-report data.

To support practical adoption, Box 1 provides a one-week microcycle sequence that translates the framework into a routine coaching workflow [1,2,3,4,5,6,7,8,9,10,11,12,13,14,15,16,17,18,19,20,21,22,23,24,25,26,27,28,29,30,31,32,33,34,35,123,124,125,126,127,128,129,130,131,132].

Box 1 **Applying LOAD-R in one weekly microcycle**

**Step 1. Define the training objective: adaptation, expression, technical quality, recovery, or competition preparation.**

**Step 2. Define the intended load and identify the key session that must be protected.**

**Step 3. Collect a minimal response set: RPE or sRPE-load, wellness/sleep, soreness, and one neuromuscular or performance marker when relevant.**

**Step 4. Classify the athlete or squad into a decision zone using convergent signals and individual baselines.**

**Step 5. Choose the action: progress, maintain, modify, deload, or recover.**

**Step 6. Re-evaluate after 24–72 h and update the baseline only when the measurement conditions are acceptable.**

In practice, decision-making emerges from the dynamic interaction between load, organism response, and context.

### Applied Microcycle Example: Operationalizing LOAD-R in Practice

To complement the procedural workflow presented in Box 1, the following example illustrates how LOAD-R can guide decision-making across a simplified weekly microcycle in a team-sport context with one competitive match. The example is intentionally simplified to illustrate decision logic rather than to prescribe a fixed training model.

This example should be read as a decision sequence rather than as a fixed-latency model. In practice, monitoring windows should be aligned with the expected time course of the stressed system: autonomic and perceptual indicators may be checked the next morning; neuromuscular responses after sprint, eccentric, resistance, or contact loads should usually be reviewed at 24 and 48 h; and delayed soreness, metabolic strain, or persistent performance suppression may require 72 h reassessment. Therefore, a modification is triggered not simply because it follows a high-load day, but because the relevant marker has exceeded measurement error, failed to rebound within the expected window, or converged with contextual risk.

Day 1 (Post-match recovery)

The athlete presents high soreness, elevated perceived fatigue, and reduced neuromuscular readiness (e.g., decreased CMJ output). The adaptive state is interpreted as transient fatigue or functional overload. The decision is to prioritize recovery through low-intensity aerobic work, mobility, and regeneration. Re-evaluation focuses on readiness rebound within 24–48 h.

Day 2 (Reintroduction)

Wellness improves, and internal load normalizes. The adaptive state transitions toward adaptive readiness. The decision is to reintroduce moderate load through technical and controlled physical work. Monitoring confirms whether recovery trends are consistent across markers.

Day 3 (High-load stimulus)

A key training session is performed. External load is high, while internal load and neuromuscular output remain stable. The adaptive state is classified as adaptive. The decision is to maintain the planned high-intensity exposure. Subsequent monitoring focuses on detecting delayed fatigue responses.

Day 4 (Fatigue monitoring and adjustment)

Slight reductions in neuromuscular readiness and increased soreness emerge. The adaptive state is interpreted as functional overload. The decision is to modify training (e.g., reduce volume or intensity) to prevent progression into maladaptive stress. Re-evaluation assesses recovery trajectory.

Day 5 (Pre-competition priming)

Readiness markers stabilize or improve. The adaptive state returns to adaptive readiness. The decision is to implement a low-volume, high-quality priming session. Monitoring confirms preparedness for competition.

Day 6 (Competition)

The match represents a high-load exposure. Performance outcome serves as the primary evaluation of preceding decisions. Post-match monitoring initiates the next LOAD-R cycle.

This example demonstrates how LOAD-R operates as a continuous adaptive loop, where training decisions emerge from the interaction between load, organism response, and adaptive-state classification, rather than from isolated variables or fixed thresholds.

Importantly, the same external load could lead to different decisions depending on athlete-specific response patterns, reinforcing the individualized and context-dependent nature of the LOAD-R framework.

## 9. Testable Predictions Emerging from the Framework

The preceding sections define the LOAD-R components, decision zones, response archetypes, and implementation levels. The predictions below, therefore, function as a validation agenda for the framework: each prediction specifies a way to test whether the proposed interpretation-to-action loop performs better than isolated metrics, fixed thresholds, or generic dashboards. A conceptual framework becomes stronger when it generates predictions that can be tested and potentially falsified.

**P1.**  Integrated prediction—In longitudinal cohort models, combined external load, internal load, readiness, and recovery features should explain more variance in 24–72 h sprint, jump, strength, endurance, or sport-task performance than load-only models.
**P2.**  Mismatch sensitivity—Lagged within-athlete analyses should show that normal external load with unusually high internal strain predicts later wellness suppression, illness flags, or performance decline earlier than either metric alone.
**P3.**  Baseline superiority—Individual rolling baselines should produce fewer false alerts and better detection of meaningful fatigue than fixed group thresholds, using SEM, CV, or smallest worthwhile change as comparison criteria.
**P4.**  Neuromuscular specificity—In strength- and power-dominant athletes, CMJ-derived markers should predict next-session sprint, jump, or maximal-intent output more strongly than generic wellness-only scores.
**P5.**  Recovery moderation—Sleep quality, soreness, HRV, and perceived recovery should moderate the load-performance relationship, with high load producing better adaptation when recovery markers remain stable.
**P6.**  State transitions—Changes between LOAD-R zones across days or microcycles should predict short-term training tolerance better than isolated single-day values.
**P7.**  Underload detection—Athletes showing high readiness, low recent load, and stagnant performance should improve after targeted increases in volume, intensity, density, or task specificity.
**P8.**  Rule-guided progression—Randomized or quasi-experimental studies should test whether LOAD-R-guided adjustment produces similar or superior performance gains with fewer maladaptive fatigue states than fixed progression.
**P9.**  Sport specificity—Monitoring batteries selected around the target performance expression should outperform generic dashboards when evaluated against sport-specific outcomes.
**P10.**  Staff communication—Decision-zone summaries should improve agreement among coaches, sport scientists, and medical staff, measured by faster action selection and higher inter-rater agreement.

Collectively, these predictions move the framework from conceptual organization toward empirical validation. The most informative studies will report measurement error, individual baselines, decision rules, state transitions, and both short-term readiness and block-level performance outcomes.

## 10. Methodological Limitations and Future Directions

This review has limitations. It is a structured narrative synthesis and does not claim exhaustive retrieval, PRISMA-level reproducibility, formal risk-of-bias scoring, or quantitative pooling. Study selection involved interpretative judgment, which may introduce selection and confirmation bias. LOAD-R also remains a conceptual framework requiring empirical validation across sports, sexes, age groups, training phases, and competitive levels. Finally, poorly selected variables could create false precision rather than better decisions, and there is also a risk that misinterpretation of multi-marker data or inconsistent measurement protocols may reduce decision accuracy.

Marker convergence is therefore a conditional principle, not a validity guarantee. Apparent agreement among variables may reflect correlated noise, redundant constructs, shared sensitivity to sleep or stress, or poor specificity for the target performance outcome. LOAD-R requires marker complementarity, reliability checks, and outcome re-evaluation before a convergent pattern is used to justify major training changes.

Future studies should test the framework rather than only apply it descriptively. Priority should be given to longitudinal designs combining external load, internal load, readiness, sleep, recovery, contextual stress, and repeated performance outcomes. Comparative studies should examine fixed progression versus LOAD-R-guided adjustment, quantify individual response, report measurement error, and predefine actions for each decision zone.

To make future validation studies auditable and comparable, Table 8 provides a reporting checklist centered on baselines, measurement error, decision rules, and outcomes.

## 11. Discussion

The present review addresses a central limitation in applied sport science: the persistent gap between athlete monitoring and actionable training decisions. Existing approaches have improved the quantification of external and internal load, but their practical value is limited when data are not converted into explicit, proportionate, and individualized actions [1,5,8,11,12,13,14].

LOAD-R builds on traditional approaches by placing adaptive-state classification between measurement and prescription. Fitness-fatigue models remain useful as conceptual foundations, but they are often difficult to operationalize in daily field settings. Workload metrics such as acute-to-chronic workload ratios can summarize exposure, but they should not be treated as universal risk thresholds. Dashboards improve visibility, but they can increase ambiguity when they do not specify action priorities [11,12,16,17,18,24,28,29,30,31,32,33,34,35].

A key contribution of the LOAD-R framework is its explicit differentiation from existing monitoring and decision-support approaches. Unlike acute-to-chronic workload ratio models, which primarily summarize load exposure and have been widely debated as risk predictors, LOAD-R does not rely on fixed ratios or universal thresholds. Instead, it interprets load in relation to organism response, individual baselines, and contextual constraints, thereby reducing the risk of misclassification based on isolated metrics [24,28,29,30,31,32,33,34,35,89,90,91,92,93,94,95].

Compared with traditional fitness-fatigue models, LOAD-R shifts the emphasis from retrospective performance modeling to real-time, field-based decision-making. While fitness-fatigue frameworks provide valuable theoretical insight into adaptation dynamics, their practical translation into daily coaching decisions is often limited. LOAD-R attempts to address this gap by introducing an intermediate adaptive-state classification that directly links observed signals to specific training actions [16,17,18,19,20].

In contrast to dashboard-based monitoring systems, which often present multiple indicators without a defined decision hierarchy, LOAD-R prioritizes signal relevance, marker complementarity, measurement reliability, and actionability. Rather than increasing informational density, the framework is intended to reduce complexity by structuring how data should be interpreted and converted into proportional training decisions [11,12,89,90,91,92,93,94,95,96,97].

Taken together, these distinctions position LOAD-R not as a competing metric or model, but as an integrative decision architecture that connects measurement, interpretation, and action within a unified adaptive loop. Its primary contribution is therefore not a new marker, formula, or technology. This shift is proposed to help reframe athlete monitoring from a descriptive process into a more operational, decision-driven system [1,2,3,4,5,6,7,8,9,10,11,12,13,14,89,90,91,92,93,94,95,96,97,98].

Its core contribution is a repeatable decision loop: define the stimulus, interpret the organism-level response, classify the adaptive state, choose a proportional action, and re-evaluate the outcome. This sequence makes the logic behind training modification visible to coaches, sport scientists, medical staff, and athletes [1,5,6,11,12,13,14,89,90,91,92,93,94,95,96,97,98].

This flexibility also creates trade-offs. The absence of rigid universal thresholds increases ecological validity and preserves coaching expertise, but it demands consistent data collection, transparent baselines, and discipline in interpreting measurement error, as well as sufficient practitioner expertise to ensure that interpretation remains consistent and contextually appropriate. A LOAD-R implementation is only as strong as the reliability of its inputs and the clarity of its predefined decisions. The present work is intended as a conceptual and translational framework, with empirical validation as a necessary next step [89,90,91,92,93,94,95,96,97,123,124,125,126,127,128,129,130,131,132].

The framework may be useful as a coach-facing scaffold and scientifically useful as a source of falsifiable predictions. Future validation should explicitly test whether LOAD-R-guided adjustment improves performance, reduces maladaptive fatigue states, improves staff agreement, or achieves similar adaptation with lower physiological and perceptual cost than fixed progression models. Its applicability may vary depending on sport-specific demands, monitoring capacity, and competitive context [23,24,25,26,27,28,29,30,31,32,33,34,35,78,79,80,81,82,83,84,85,86,87,88,110,111,112,113,114,115,116,117,118,119,120,121,122].

Accordingly, LOAD-R should be regarded as a testable decision scaffold rather than as a validated superiority claim; its practical value must be established against alternative monitoring and coaching workflows.

The added practical value of LOAD-R is therefore best understood as a communication and governance tool for training decisions. It makes explicit who collected the signal, how the signal was interpreted, which contextual constraints were considered, what action was selected, and how the decision was checked afterward. This may improve staff agreement and reduce arbitrary reactions to isolated alerts, but it cannot remove the need for sport-specific expertise, careful variable selection, or prospective validation against real performance and health outcomes.

### Practical Applications

Practitioners should treat monitoring data as action cues rather than independent verdicts. The minimum sequence is: define the session objective; compare planned and actual load; interpret response against individual baseline and measurement error; classify adaptive state; select an action; and re-check the outcome after 24–72 h [1,5,8,11,12,13,14,89,90,91,92,93,94,95,96,97,98]. When possible, the 24–72 h re-check should be marker-specific: acute perceptual/autonomic indicators can be reviewed early, whereas neuromuscular and soreness responses may require repeated 24-, 48-, and 72 h checks before a strong decision is made.

Operationally, LOAD-R should favor small, reversible adjustments when signals are uncertain, protect key sessions when readiness and performance are stable, and prioritize recovery or medical/contextual review when suppressed readiness, high internal strain, and declining output converge. This ensures that monitoring remains actionable, scalable, and directly aligned with training effectiveness rather than data accumulation [55,56,57,61,62,63,64,65,78,79,80,81,82,83,84,85,86,87,88,110,111,112,113,114,115,116,117,118,119,120,121,122].

## 12. Conclusions

This review reframes athlete monitoring as an adaptive decision-support process rather than a passive data-collection exercise. The proposed LOAD-R framework integrates load exposure, organism response, adaptive-state classification, decision-making, and re-evaluation into a unified loop.

Its primary contribution is organizational: it does not require new variables, but it clarifies how existing monitoring information should be interpreted and converted into training actions. In doing so, it may help practice move beyond single indicators, fixed thresholds, and dashboard alerts.

From a practical perspective, LOAD-R supports scalable implementation across amateur, university, and high-performance settings by emphasizing decision clarity over data complexity. From a scientific perspective, it provides testable predictions for future longitudinal and intervention-based research.

The effectiveness of the framework will ultimately depend on empirical validation across sports, contexts, and populations. Its immediate value is proposed to lie in providing a clear, individualized, and context-aware architecture for adaptive training and load management.

## Figures and Tables

**Table 1:** Evidence domains and decision value in adaptive training.

Evidence Domain	Primary Contribution	Common Failure When Isolated	Function in LOAD-R
External load	Describes the mechanical or task stimulus applied to the athlete.	High values may be over-interpreted as inherently dangerous or inherently productive.	Defines the L component: what the athlete was asked to absorb.
Internal load	Captures the perceived or physiological cost of completing the session.	May be inflated by non-training stress, sleep loss, or illness if the context is ignored.	Begins the O component: how the athlete experienced the stimulus.
Readiness and fatigue	Indicates whether neuromuscular, perceptual, or autonomic status is stable, elevated, or suppressed.	Single markers can be noisy, insensitive, or sport-specific.	Supports A: classification of adaptive state.
Recovery and context	Places performance output in the broader 24- to 72-h recovery architecture.	Recovery signals can be mistaken for weakness rather than capacity constraints.	Constrains D: the magnitude of progression or reduction.
Performance outcome	Tests whether the decision improved task quality or adaptation.	The outcome is often checked too late or detached from the preceding state.	Completes R: re-evaluation and baseline updating.

Notes: This table summarizes the logic used to organize the narrative synthesis. Evidence domains were not treated as competing tools but as complementary layers in a decision system [1,2,3,4,5,6,7,8,9,10,11,12,13,14,15,16,17,18,19,20,21,22,23,24,25,26,27,28,29,30,31,32,33,34,35].

**Table 2:** Monitoring variables and their applied interpretation.

Domain	Representative Variables	Practical Interpretation	Minimum Field Implementation
External load	Distance, high-speed running, accelerations, decelerations, sprint volume, tonnage, sets, reps, velocity loss.	Defines the imposed stimulus and allows comparison between planned and actual exposure.	Training diary plus sport-relevant load metric.
Internal load	RPE score, session RPE, sRPE-load (RPE × duration), heart rate, TRIMP, perceived session difficulty.	Describes the athlete-specific cost of the same external stimulus.	Session RPE collected 15–30 min post-session; compute sRPE-load only when duration is multiplied explicitly.
Neuromuscular readiness	CMJ height, flight time:contraction time, RSI-modified, jump power, force-time variables.	Identifies acute changes in power, stiffness, or neuromuscular function.	Standardized CMJ protocol 2–3 times weekly.
Recovery and wellness	Sleep, soreness, fatigue, stress, mood, perceived recovery, HRV.	Places training tolerance inside broader recovery capacity.	Short wellness form plus sleep duration/quality.
Performance outcome	Sprint, jump, strength, endurance, and sport-specific task quality.	Determines whether the monitoring-guided decision improved the target.	Weekly or block-level performance check.

Notes: Representative variables are examples, not a mandatory battery. RPE, session RPE, and sRPE-load are related but not interchangeable. Selection should be sport-specific, reliable, repeatable, and linked to decisions [36,37,38,39,40,41,42,43,44,45,46,47,48,49,50,51,52,53,54,55,56,57,58].

**Table 3:** LOAD-R components and operational questions.

Component	Operational Question	Primary Data Sources	Default Decision Relevance
**L—Load**	What stimulus was planned and what was actually completed?	Session plan, GPS, training diary, tonnage, velocity, duration, sRPE-load.	Defines whether progression, maintenance, or reduction is even plausible.
**O—Organism response**	How did the athlete absorb the stimulus?	RPE, HR/HRV, CMJ, wellness, soreness, sleep, mood, and technical quality.	Identifies tolerance, strain, or mismatch.
**A—Adaptive state**	What state best explains the current response pattern?	Individual baseline, marker convergence, trend, context, measurement error.	Classifies adaptive, functional overload, underload, uncertainty, or maladaptation.
**D—Decision**	What should be changed today or this week?	Training objective, priority session, competitive calendar, and athlete state.	Progress, maintain, modify, deload, recover.
**R—Re-evaluation**	Did the decision improve performance or reduce cost?	Next-day readiness, weekly performance, repeated tests, injury/illness flags.	Updates baselines and improves future decisions.

Notes: LOAD-R is intended as a flexible architecture rather than a universal algorithm. Each component should be populated with variables appropriate to the sport, competitive level, and resources available [1,2,3,4,5,6,7,8,9,10,11,12,13,14,15,16,17,18,19,20,21,22,73,74,75,76,77,78,79,80,81,82,83,84,85,86,87,88].

**Table 4:** Decision-zone interpretation for adaptive load management.

Zone	Load/Readiness Pattern	Interpretation	Default Action	Risk If Misread
Green—Adaptive	Moderate/high load with stable or high readiness.	The athlete is tolerating the stimulus.	Maintain or progress according to plan.	Underloading a prepared athlete.
Yellow—Functional overload	High load with mild, expected readiness suppression.	Short-term stress may be productive if planned.	Maintain briefly; monitor closely.	Extending overload beyond tolerance.
Red—Maladaptive stress	High load with persistent readiness suppression and performance decline.	Fatigue accumulation or unresolved stress is likely.	Reduce intensity/volume; prioritize recovery.	Ignoring early warning signs.
Blue—Underload	Low load with high readiness and flat performance.	Stimulus may be insufficient.	Increase load, density, or specificity.	Mistaking freshness for optimal adaptation.
Gray—Uncertain	Variable or conflicting signals.	Measurement noise, context, or poor protocol may dominate.	Repeat measure; check sleep, stress, illness, technique.	Acting aggressively on unreliable data.

Notes: Zone categories are decision aids. They should be interpreted relative to individual rolling baselines, known measurement error, sport context, session priority, and athlete history rather than as universal thresholds, and should not be exported directly across sports without local validation [23,24,25,26,27,28,29,30,31,32,33,34,35,89,90,91,92,93,94,95,96,97,98].

**Table 5:** If-then rules for coach-facing training adjustment.

Condition	Likely Interpretation	Training Decision	Follow-Up Question
High external load + stable readiness + stable output	Productive loading and adequate tolerance.	Maintain planned progression.	Is the next key session protected?
High load + reduced CMJ +high soreness	Neuromuscular fatigue after high mechanical stress.	Reduce sprint/power exposure or convert to technical work.	Does readiness rebound within 24–72 h?
Normal external load +unusually high sRPE	Internal strain mismatch.	Check sleep, stress, illness, nutrition, and heat exposure.	Is this athlete usually honest and consistent with RPE?
Low load + high readiness +flat performance	Insufficient stimulus or poor specificity.	Increase load, intensity, or task specificity.	Is the athlete protected too much?
Low load + low readiness	Non-training stress or recovery debt.	Prioritize recovery and contextual assessment.	What happened outside training?
Stable load + rising RPE across the week	Accumulating fatigue or reduced tolerance.	Reduce density or insert recovery.	Is performance also declining?
Suppressed readiness before the low-priority session	Recovery opportunity.	Convert to a regenerative or technical session.	Can the high-value session be protected?
Suppressed readiness before the key session	Risk of low-quality output.	Modify objective or extend warm-up; avoid maximal exposure if convergent signals are poor.	Is the competition calendar forcing risk?
Performance improves after deload	Accumulated fatigue confirmed.	Adjust future overload duration.	Was the previous block too long or too dense?
Performance does not improve after deload	A deeper issue may be present.	Assess injury, illness, sleep, nutrition, and psychological load.	Is referral or medical review needed?

Notes: Rules are deliberately conditional. They translate common monitoring patterns into actions while preserving coaching judgment and contextual interpretation [1,2,3,4,5,6,7,8,9,10,11,12,13,14,15,16,17,18,19,20,21,22,23,24,25,26,27,28,29,30,31,32,33,34,35,89,90,91,92,93,94,95,96,97,98].

**Table 6:** Response archetypes and monitoring priorities.

Archetype	Typical Pattern	Primary Risk	Monitoring Priority	Coaching Response
Fast adapter	Readiness stable; performance improves during progressive load.	Under-challenging the athlete.	Track progression without excessive testing burden.	Progress cautiously and protect recovery.
Fatigue accumulator	Readiness declines; RPE rises; output falls after repeated loading.	Continuing overload beyond useful adaptation.	CMJ, soreness, sleep, RPE trend, next-day performance.	Deload, redistribute intensity, or reduce density.
Underloaded athlete	Readiness high; load low; performance stagnates.	Comfortable non-adaptation.	Performance trend and stimulus specificity.	Increase stimulus or change training content.
Context-stressed athlete	Load moderate; wellness poor; readiness variable.	Misattributing life stress to poor motivation.	Sleep, stress, mood, academic/workload, and illness flags.	Modify load and address context.
Unstable responder	Large day-to-day variability across markers.	Overreacting to noisy signals.	Protocol consistency and repeated measurements.	Stabilize the schedule and measurement conditions.

Notes: Archetypes are interpretative aids, not diagnostic labels. Athletes may move between archetypes across phases, sports, and competitive demands [99,100,101,102,103,104,105,106,107,108,109,110,111,112,113,114,115,116,117,118,119,120,121,122].

**Table 7:** Implementation levels for LOAD-R in applied sport settings.

Level	Tools	Best Suited for	Decision Strength	Main Constraint
Low-cost	RPE/sRPE-load, wellness, sleep log, training diary, periodic CMJ or jump app.	Amateur clubs, university teams, small performance programs.	Strong if the collection is consistent and actions are explicit.	Limited precision and possible self-report bias.
Moderate	Jump mat, HR monitor, structured dashboard, simple field tests.	Academies, semi-professional teams, and developing high-performance programs.	Allows weekly state tracking and more reliable trends.	Requires staff time and protocol discipline.
High-performance	GPS/LPS, force plate, HRV, sleep wearable, integrated AMS.	Elite team sports and professional programs.	Supports individual response modeling and staff communication.	Data overload and false precision.
Research-grade	Longitudinal modeling, biomarker sampling, advanced statistics, and validation design.	Applied research and framework testing.	Can test predictions and refine thresholds.	Higher cost and complexity.

Notes: Tool level does not determine decision quality. The most important requirement is that variables are reliable, context-specific, and tied to predefined actions; in lower-resource settings, subjective scales also require familiarization and calibration before they are used for high-stakes decisions [123,124,125,126,127,128,129,130,131,132,133,134,135,136,137,138,139].

**Table 8:** Reporting and validation checklist for future LOAD-R studies.

Design Element	Rationale	Recommended Minimum Practice
Individual baseline	Group thresholds can hide meaningful within-athlete change.	Report baseline window, reliability, and decision threshold.
Measurement error	Small changes may be noise.	Report ICC, CV, SEM, or the smallest worthwhile change where possible.
Training objective	The same marker has a different meaning across session goals.	State whether the goal is adaptation, expression, recovery, or competition specificity.
Contextual stress	Sleep, soreness, illness, travel, or academic load can change tolerance.	Collect a concise context/wellness measure.
Decision rule	Monitoring without action is incomplete.	State what action was taken when thresholds or zones were reached.
Outcome check	The decision must be evaluated.	Report next-day readiness and block-level performance outcomes.
Sport specificity	Generic dashboards may miss target performance.	Justify variable selection based on sport and training phase.

Notes: The checklist is intended to improve interpretability, not to impose a rigid reporting standard. It aligns monitoring design with the requirements of decision support and future validation [89,90,91,92,93,94,95,96,97,98,99,100,101,102,103,104,105,106,107,108,109,110,111,112,113,114,115,116,117,118,119,120,121,122,123,124,125,126,127,128,129,130,131,132].

## Data Availability

No new data were created or analyzed in this study. Data sharing is not applicable to this article.

## Abbreviations

ACWR	acute-to-chronic workload ratio
AMS	athlete management system
CMJ	countermovement jump
GPS	global positioning system
HR	heart rate
HRV	heart rate variability
LOAD-R	Load, Organism response, Adaptive state, Decision, Re-evaluation
RPE	rating of perceived exertion
RSI	reactive strength index
SEM	standard error of measurement
sRPE	session rating of perceived exertion; when multiplied by duration, reported here as sRPE-load
TRIMP	training impulse
